# Levamisole, as a viral vaccine adjuvant, induces robust host defense through the modulation of innate and adaptive immune responses

**DOI:** 10.3389/fmicb.2024.1493561

**Published:** 2025-01-08

**Authors:** Gang Sik Kim, Dong Yun Kwak, Hyeong Won Kim, Seokwon Shin, Mi-Kyeong Ko, Seong Yun Hwang, So Hui Park, Dong Hyeon Kim, Jong-Hyeon Park, Su-Mi Kim, Min Ja Lee

**Affiliations:** Center for Foot-and-Mouth Disease Vaccine Research, Animal and Plant Quarantine Agency, Gimcheon-si, Republic of Korea

**Keywords:** levamisole, vaccine adjuvant, host defense, immunomodulation, innate and adaptive immunity

## Abstract

**Introduction:**

An effective vaccination policy must be implemented to prevent foot-and-mouth disease (FMD). However, the currently used vaccines for FMD have several limitations, including induction of humoral rather than cellular immune responses.

**Methods:**

To overcome these shortcomings, we assessed the efficacy of levamisole, a small-molecule immunomodulator, as an adjuvant for the FMD vaccine. We conducted *in vitro* studies using murine peritoneal exudate cells (PECs) and porcine peripheral blood mononuclear cells (PBMCs) and *in vivo* studies using mice (experimental animals) and pigs (target animals). We evaluated levamisole-mediated modulation of the innate and adaptive immune responses; early, mid-term, and long-term immune-inducing effects; modes of action; and host defense against viral infection.

**Results:**

Levamisole treatment promoted IFNγ secretion in murine PECs and porcine PBMCs. Additionally, it induced robust and long-lasting immune responses by eliciting high antibody titers and high virus-neutralizing antibody titers. By activating downstream signaling pathways of various pattern-recognition receptors, levamisole stimulated the expression of multiple cytokines and costimulatory molecules. Owing to these immunostimulatory effects, levamisole elicited host defense against viral infections in pigs. Our findings demonstrate the potential of levamisole as an immunostimulatory agent.

**Discussion:**

The results also indicate that levamisole, as an adjuvant for animal vaccines, can elicit robust innate and adaptive immune responses, thereby enhancing host defense against viral infections. This study provides a promising approach for the development of improved FMD vaccine strategies in the future.

## 1 Introduction

Foot-and-mouth disease (FMD) is a highly contagious and major infectious viral disease in the livestock industry that affects cloven-hoofed animals. It is fatal in young animals because it causes myocarditis (Grubman and Baxt, 2004). The characteristic clinical symptoms of FMD include high fever, followed by the formation of blisters inside the mouth and near the hooves that burst and cause lameness; in severe cases, the hooves fall off. FMD virus (FMDV) can easily spread through contact with contaminated animals or feed (Arzt et al., 2011; Stenfeldt et al., 2014; Fukai et al., 2015b; Casey-Bryars et al., 2018). Considerable efforts, including vaccination and strict monitoring, are required to control it; in case of infection, strict trade restrictions, quarantine, and culling of both infected and non-infected animals are needed. The high genetic variability of FMDV limits the efficacy of vaccination, which considerably varies between and even within serotypes; in particular, there is no cross-protection among serotypes (Mason et al., 2003; Grubman and Baxt, 2004). Vaccination policies have been implemented in several endemic countries to effectively prevent FMD (Rodriguez and Grubman, 2009; Casey-Bryars et al., 2018). The efficacy of FMD vaccines has improved in terms of relatively high antibody (Ab) titers. However, the current FMD vaccines have several limitations. FMD vaccines act by inducing humoral immunity using an oil emulsion as an adjuvant. As such, the induction of Ab titers is slow, and host defense against viral infection is difficult in the early stages of vaccination.

To overcome these limitations, we have been conducting research on strategies to strengthen the immunogenicity of inactivated antigens derived from the FMDV itself (Lee et al., 2020; Lee et al., 2022) and on developing new adjuvants, such as immunostimulants and immunomodulators (Lee et al., 2019; Jo et al., 2021; Kim et al., 2023a; Kim et al., 2023b; Ko et al., 2023; Shin et al., 2023; Kim et al., 2024a; Kim et al., 2024b). An adjuvant is a substance that does not show immunogenicity on its own but strengthens the immune response through physical and chemical binding to an antigen. Adjuvants enhance immune responses to antigens present in the vaccine (Brewer, 2006; Kuroda et al., 2013). Commercial FMD vaccine adjuvants commonly include (1) mineral oil-based emulsions, including Marcol 52^™^, Montanide^™^ ISA 50, and Montanide^™^ ISA 206, and (2) antigen-delivery systems or immunostimulants, such as aluminum gel or saponins. FMD vaccine is manufactured as an oil emulsion to ensure that the antigen is released slowly after vaccination, and the antibody titer is sustained for a long time. Saponins are naturally occurring glycosides that are used in various veterinary and human vaccines. Aluminum-based adjuvants were introduced in 1925 by Glenny et al. (1926) and are still considered the gold standard. These include aluminum hydroxide [Al(OH)_3_] and alum. Al(OH)_3_ is the most widely used adjuvant. When an antigen is absorbed by Al(OH)_3_, it aggregates on the surface and inside the Al(OH)_3_-based adjuvant particles, which helps maintain the physiochemical properties of the antigen. Al(OH)_3_ not only protects antigens by absorbing them to form a depot but also acts as an antigen delivery system to induce a Th2 immune response. Quil-A, a saponin mixture, has been commonly used as an adjuvant because of its superior adjuvanticity to activate dendritic cells (DCs), strengthen Ab responses, and stimulate Th1 cells and cytotoxic T lymphocyte (CTL) immune responses (Marciani, 2018; Lacaille-Dubois, 2019). However, saponins do not form depots and are toxic when administered in excess.

To achieve a potent immune response and complete host defense *via* vaccination, efforts are needed to discover new adjuvants (immunostimulants and immunomodulators) that can induce innate and adaptive immunity. Levamisole, a small synthetic molecule, was included in the World Health Organization list of major drugs in 1988 owing to its broad-spectrum activity, safety, and relatively low cost (World Health Organization, 2019). It is an imidothiazole derivative that has various applications in the medical field. Levamisole is commonly used as a free-base or hydrochloride salt. Levamisole has long been utilized as an anthelmintic for livestock and is used as a monotherapy or adjunct therapy for a variety of diseases such as colon cancer and dermatological problems. Levamisole is used as an allotherapy in countries where lumpy skin disease is endemic (Huskisson et al., 1976; Mutch and Hutson, 1991; Chlebowski et al., 1994; Scheinfeld et al., 2004). Levamisole performs broad immunomodulatory functions by activating DCs and enhancing the production of cytokines, including interleukin (IL)-12 and IL-10. Levamisole-mediated anti-inflammatory action occurs due to the inhibition of the expression of tumor necrosis factor (TNF)*α* and IL-6. Levamisole enhances immune responses by stimulating DCs to mature peripheral DCs and inducing the expression of interferon (IFN)*γ* (Renoux, 1980). However, the detailed mechanisms of levamisole-mediated immune responses, when used with vaccines for controlling veterinary diseases, are not clear. As levamisole is quickly metabolized *in vivo* and regulates the host immune response, a moderate concentration of this compound is sufficient as an animal vaccine (Campillo et al., 2022). Despite numerous reports on levamisole, its use as an adjuvant in FMD vaccines has not been reported. To overcome the shortcomings of the commercial FMD vaccines, we developed a next-generation FMD vaccine that induces robust host defense by eliciting innate and adaptive immunity using levamisole as an adjuvant and evaluated its efficacy through *in vitro* and *in vivo* studies in mice and pigs.

## 2 Materials and methods

### 2.1 Levamisole

Levamisole hydrochloride was purchased from Sigma-Aldrich (Cat Number: Y0000047; St. Louis, MO, United States). Levamisole (15 mg) was dissolved in 1 mL of physiological water for use.

### 2.2 Cells, virus, and purification of inactivated viral antigens

Baby hamster kidney [BHK-21 (C-13); American Type Culture Collection (ATCC), Manassas, VA, United States], fetal porcine kidney (LF-BK; Plum Island Animal Disease Center, Orient, NY, United States), and fetal goat tongue epithelium [ZZ-R 127; the Collection of Cell Lines in Veterinary Medicine (CCLV), Friedrich-Loeffler-Institut, Greifswald-Insel Riems, (Germany)] cells were cultured as described previously (Kim et al., 2023a; Kim et al., 2024a). FMDV antigens (O PA2 and A YC) were produced as explained previously [12]. Sixteen hours after infection, the viruses were inactivated using two doses of binary ethyleneimine (0.003 mM) for 24 h and concentrated using polyethylene glycol 6000 (Sigma-Aldrich) (Bahnemann, 1975). Antigens were purified using the sucrose density gradient method (15–45%), followed by ultracentrifugation. Viral antigen (146S particles) was quantified by measuring the absorbance at 259 nm using a spectrophotometer (Hidex, Turku, Finland). An inactivation test using ZZ-R 127 and BHK-21 cells confirmed that no live viruses were present in the inactivated supernatants. All FMDV-related experiments were conducted in a biosafety level 3 (BSL-3) facility.

### 2.3 Preparation of test vaccine

In the mouse experiment, the vaccine composition of the positive control (PC) group was as follows: antigen types O (O PA2; 0.375 μg/dose) and A (A YC; 0.375 μg/dose), 15 μg/dose Quil-A (InvivoGen, San Diego, CA, United States), 10% Al(OH)_3_, and ISA 206 (Seppic, Paris, France; 50% w/w). The experimental (Exp) group received vaccines with the same composition as that for the PC group, with the addition of 100 μg levamisole/dose. One dose comprised a total volume of 100 μL.

In the pig experiment, the vaccine formula for the PC group was as follows: antigen types O (15 μg/dose) and A (15 μg/dose), 150 μg/dose Quil-A, 10% Al(OH)_3_, and ISA 206 (50% w/w). The Exp group received vaccines with the same formula as that for the PC group, with the addition of 1 mg levamisole/dose. One dose comprised a total volume of 1 mL.

### 2.4 Mice and pigs

Mice (C57BL/6, females, 6–7 weeks old) were obtained from KOSA BIO Inc. (Gyeonggi-do, Korea), and pigs (Landrace, 8–9 weeks old) were procured from BARON BIO Inc. (Gyeongsandbuk-do, Korea). All the animals were acclimated to the accommodation environment for 1 week prior to the experiments in a dedicated pathogen-free ABSL-3 facility at the APQA. The experiments were performed in compliance with relevant regulations [established by the institution and authorized by the Ethics Committee of the APQA (accreditation numbers: IACUC-2022-659 and IACUC-2023-719)].

### 2.5 Peritoneal exudate and peripheral blood mononuclear cell isolation

Naïve mice (*n* = 20) were euthanized *via* CO_2_ inhalation. The abdominal cavity of mice was carefully flushed with Hanks’ buffered saline solution (HPBS; Hyclone, Logan, UT, United States) and centrifuged (400 × g, 10 min, 4°C). Peripheral blood mononuclear cells (PBMCs) were purified from the pigs [*n* = 3 for the evaluation of IFNγ secretion using enzyme-linked immunosorbent spot (ELISpot) assay and *n* = 5–6/group for validation of gene expression using quantitative reverse transcription-polymerase chain reaction (qRT-PCR)] that tested negative for FMD antibodies in donor confirmation, following the purification process for whole blood (Kim et al., 2023b). Whole blood was independently collected in heparin tubes (BD Biosciences, Franklin Lakes, NJ, United States). PBMCs were isolated with Lymphoprep^™^ (StemCell Technologies, Vancouver, Canada). The remaining red blood cells (RBCs) were eliminated using a cold RBC lysis buffer (BioLegend, San Diego, CA, United States). The isolated peritoneal exudate cells (PECs) and PBMCs were counted and cultured in RPMI-1640 medium (Gibco, San Francisco, CA, United States). All cells were used immediately after isolation.

### 2.6 Levamisole-mediated cell viability and IFNγ ELISpot assay *in vitro*

BHK-21 (C-13), LF-BK, and ZZ-R 127 cell lines, murine PECs, and porcine PBMCs were used for cell viability assays. All cells (1 × 10^5^ cells/well) were seeded in a 96-well microplate and incubated for 1 h under 5% CO_2_ at 37°C. After incubation, the culture medium was changed, and the cells were treated with levamisole (0, 0.625, 1.25, 2.5, or 5 μg/mL) for 4 h. Cell viability was determined using the MTS-based assay in accordance with the manufacturer’s instructions (Promega, Madison, WI, United States). An ELISpot assay was conducted using the IFNγ immunospot kit (R&D Systems, Minneapolis, MN, United States) to analyze the IFNγ secretion induced by levamisole, with or without inactivated FMDV (O PA2 or A YC) antigen, according to the manufacturer’s instructions. Briefly, the isolated murine PECs (5 × 10^5^ cells/well) or porcine PBMCs (5 × 10^5^ cells/well) were stimulated with 2 μg/mL (final concentration) of inactivated FMDV antigens, either without or in combination with 0.625, 1.25, 2.5, and 5 μg/mL levamisole sequentially. PBS and the antigen alone were used as the negative control (NC) and PC, respectively. The results were confirmed using an ImmunoSpot ELISpot reader (Autoimmun Diagnostika GmbH, Strasburg, Germany). Data are presented as the number of spot-forming cells.

### 2.7 Levamisole alone-mediated host defense against FMDV infection in mice

Prior to investigating the adjuvanticity of levamisole, we evaluated the host defense mediated by levamisole alone (without the FMDV antigen) against FMDV type O [O/VET/2013 (ME-SA topotype, GenBank Accession No. MF947143.1)] or type A [(A/Malay/97, SEA topotype, GenBank Accession No. KJ933864)] infection in mice (*n* = 5/group). Mice were administered 100 μg levamisole in a total volume of 100 μL. Mice were administered an intramuscular (IM) injection [0 days post-injection (dpi)] and challenged with FMDV (100 LD_50_ of O/VET/2013 or 100 LD_50_ of A/Malay/97) *via* intraperitoneal (IP) injection at 3 or 7 dpi. The survival rates and body weights were monitored for up to 7 days post-challenge (dpc).

### 2.8 Assessment of the safety of FMD vaccine containing levamisole in mice

The safety of the FMD vaccine containing levamisole was evaluated in mice as described previously (Kim et al., 2023a; Kim et al., 2024a). Mice (*n* = 5/group) were vaccinated with a five-fold (500 μL) dose of the vaccine. Mice were vaccinated *via* the IP route (0 dpi). The survival rates and changes in body weight were evaluated up to 7 dpi.

### 2.9 Host defense mediated by FMD vaccine containing levamisole as an adjuvant in mice

Host defense against viral infections was assessed in mice vaccinated with the vaccine. Mice (*n* = 5/group) were vaccinated *via* IM injection [0 days post-vaccination (dpv)] and challenged with FMDV (100 LD_50_ of O/VET/2013 or 100 LD_50_ of A/Malay/97) *via* IP injection at 7 dpv. The survival rates and body weights were monitored for up to 7 dpc.

### 2.10 Early, mid-term, and long-term immunity mediated by FMD vaccine containing levamisole as an adjuvant in mice

We assessed the efficacy of levamisole as an FMD vaccine adjuvant in eliciting innate and adaptive (cellular and humoral) immunity in mice. Mice (*n* = 5/group) were immunized with the test vaccine *via* the IM route, and blood samples were collected at 0, 7, 28, 56, and 84 dpv for serological analysis. Serum samples were stored at −80°C until analysis.

### 2.11 Early, mid-term, and long-term immunity mediated by FMD vaccine containing levamisole as an adjuvant in pigs

Early, mid-term, and long-term immunity to the test vaccine was evaluated in pigs (*n* = 5–6/group) using a previously described experimental protocol (Kim et al., 2023a; Kim et al., 2024a). For serological analysis, sera were collected from the vaccinated pigs at 0, 7, 14, 28, 56, and 84 dpv. After the first vaccination, a second vaccination was performed at 28 dpv using the same route. Serum samples were stored at −80°C until analysis.

### 2.12 Serological assay

#### 2.12.1 Enzyme-linked immunosorbent assay of the structural protein

To evaluate structural protein (SP) Abs in sera, we utilized the PrioCheck^™^ FMDV type A (Prionics AG, Schlieren, Switzerland) and VDPro^®^ FMDV type O (Median Diagnostics, Gangwon-do, Korea) kits. Absorbance was measured at a wavelength of 450 nm and converted to percent inhibition (PI) values. The animals were classified as positive for antibodies at PI ≥50% for the PrioCheck^™^ FMDV kit and PI ≥40% for the VDPro^®^ FMDV kit.

#### 2.12.2 Virus neutralizing test

The virus neutralizing (VN) test was conducted according to protocols specified by the World Organization for Animal Health (WOAH). Briefly, serum was heat-inactivated and then diluted. Thereafter, 50 μL TCID_50_ FMDV (O PA2, O JC, A YC, or A GP) was added and incubated for 1 h. A 50 μL volume of LF-BK cells (1 × 10^4^ cells/well) was added to each well and cultured for 3 days. Subsequently, cytopathic effects were confirmed in each well (Fowler et al., 2010; Fukai et al., 2015a).

#### 2.12.3 Immunoglobulin subtype (IgG, IgA, and IgM) ELISA

To evaluate antigen-specific antibodies [immunoglobulin (Ig) subtype], enzyme-linked immunosorbent assay (ELISA) was performed for porcine IgM, IgA, and IgG (Bethyl Laboratories Inc., Montgomery, TX, United States) using serum samples as per the manufacturer’s guidelines. The absorbance at 450 nm was read using a spectrophotometer (Kim et al., 2023a; Kim et al., 2024a).

### 2.13 RNA isolation, cDNA synthesis, and qRT-PCR

We investigated the mechanism of immune response elicited by the FMD vaccine containing levamisole using a protocol described previously (Kim et al., 2023a; Kim et al., 2024a). Total RNA was extracted using TRIzol^®^ reagent (Thermo Fisher Scientific, Waltham, MA, United States) and Rneasy Mini Kit (QIAGEN, Valencia, CA, United States) as per the manufacturers’ guidelines. Complementary DNA (cDNA) was prepared *via* reverse transcription using the GoScript Reverse Transcription System (Promega) according to the manufacturer’s guidelines. The synthesized cDNAs were amplified *via* qRT-PCR on a Bio-Rad iCycler using the iQ SYBR Green Supermix (Bio-Rad, Hercules, CA, United States). The qRT-PCR results were normalized using the measured HPRT (reference gene) levels. The primers used are listed in Supplementary Table S1.

### 2.14 Evaluation of host defense against FMDV infection induced by the test vaccine

A challenge experiment was performed (*n* = 3–4/group) to evaluate whether the test vaccine induced host defense. At 28 dpv, all groups were infected with FMDV types O (O/SKR/JC/2014; 10^5^ TCID_50_/100 μL) *via* intradermal injections into the soles of the feet. Observation of clinical symptoms and collection of oral swab samples (BD™ Universal Viral Transport Kit; BD Biosciences) were performed daily during the challenge period. Serum was prepared from blood collected in Vacutainer serum tubes (BD Biosciences) at 0, 2, 4, 6, and 8 dpc. RNA was extracted from oral swabs and serum using the QIAcube^®^ HT Pathogen Kit (QIAGEN, Hilden, Germany), according to the instructions provided by the manufacturer. RT-PCR was conducted using the FMDV Real-Time RT-PCR Master Mix Kit (Bioneer, Daejeon, Korea), according to the manufacturer’s guidelines (Kim et al., 2023a; Kim et al., 2024a).

### 2.15 Statistical analysis

Unless otherwise specified, all data are presented as the mean ± SEM. Survival curves were drawn using the Kaplan–Meier method, and differences analyzed using the log-rank sum test. Statistical differences between groups were calculated using Tukey’s or Dunnett’s *post-hoc* tests and one-way or two-way analysis of variance. Statistical significance is indicated by ^*^p < 0.05, ^**^p < 0.01, ^***^p < 0.001, and ^****^p < 0.0001. All data were analyzed using GraphPad Prism 10.0.2 (GraphPad, San Diego, CA, United States).

## 3 Results

### 3.1 Levamisole elicits innate and adaptive (cellular) immunity *via* IFNγ expression in PECs and PBMCs *in vitro* and drives adjuvanticity

**Figure 1:**
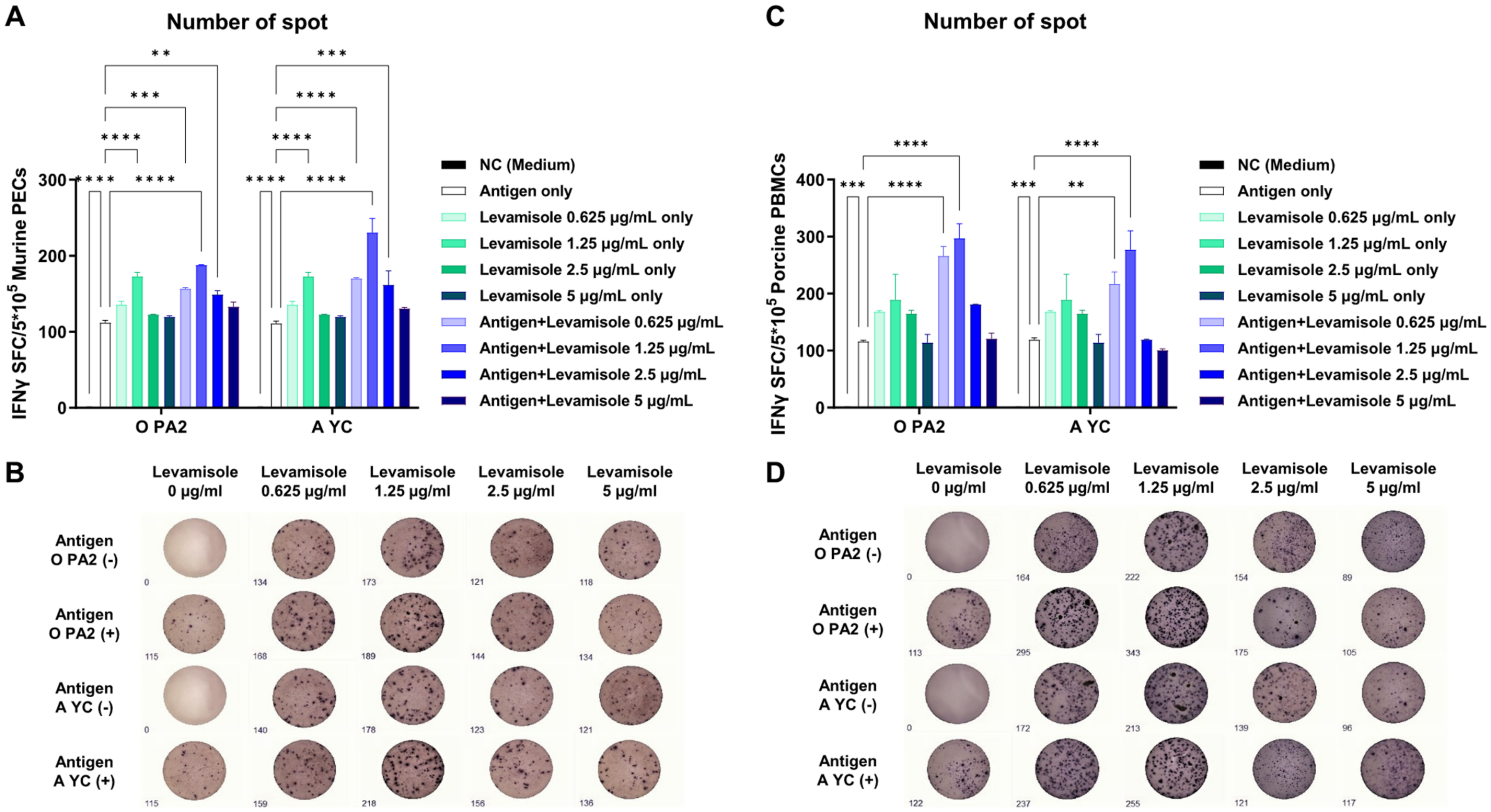
Levamisole mediates innate immune response *via* IFNγ secretion in murine PECs and porcine PBMCs. To evaluate the innate immune response to levamisole, with or without inactivated foot-and-mouth disease virus (FMDV) type O (O PA) or A (A YC) antigen, IFNγ secretion was determined using the enzyme-linked immunosorbent spot (ELISpot) assay. **(A)** Number of IFNγ-secreting cell spots in murine peritoneal exudate cells (PECs). **(B)** Images of IFNγ-secreting murine PECs. **(C)** Number of IFNγ-secreting cell spots in porcine peripheral blood mononuclear cells (PBMCs). **(D)** Images of IFNγ-secreting porcine PBMCs. Data are presented as the mean ± SEM of the number of spot-forming cells (SFCs) obtained from triplicate measurements (*n* = 3/group). Statistical analyzes were conducted using a one-way ANOVA followed by Tukey’s *post-hoc* test. ^*^p < 0.05, ^**^p < 0.01, ^***^p < 0.001, and ^****^p < 0.001.

Prior to conducting a series of experiments using levamisole, we confirmed that levamisole treatment was non-cytotoxic at concentrations ≤5 μg/mL in BHK-21 (C-13), LF-BK, ZZ-R 127, PECs, and PBMCs (Supplementary Figures S1A–E). The IFNγ secretion in PECs and PBMCs was significantly higher in the Exp group administered FMDV (O PA2 or A YC) antigens with levamisole than in the antigen-only group. In particular, IFNγ secretion was the highest in PECs and PBMCs when 1.25 μg of levamisole was administered together with the antigen (Figures 1A–D).

### 3.2 FMD vaccine containing levamisole as an adjuvant elicits potent host defense in the early stage of viral infection in mice

Prior to evaluating the host defense elicited by FMD vaccine containing levamisole, we investigated the host defense mediated by levamisole alone against FMDV infection (O/VET/2013 or A/Malay/97) in mice. Although levamisole induced significant IFNγ secretion in the experiment described in the previous section, levamisole alone did not elicit host defense against viral infection (Supplementary Figures S2A–I). To assess the safety of the vaccine containing levamisole, mice were administered 500 μL of FMD vaccine containing levamisole *via* the IP route, and their survival rate and changes in body weight were monitored until 7 dpv. The mice showed a 100% survival rate and no changes in body weight, which indicated the safety of the FMD vaccine containing levamisole as an adjuvant (Supplementary Figures S3A,B). Based on these results, we evaluated the adjuvanticity of levamisole.

**Figure 2:**
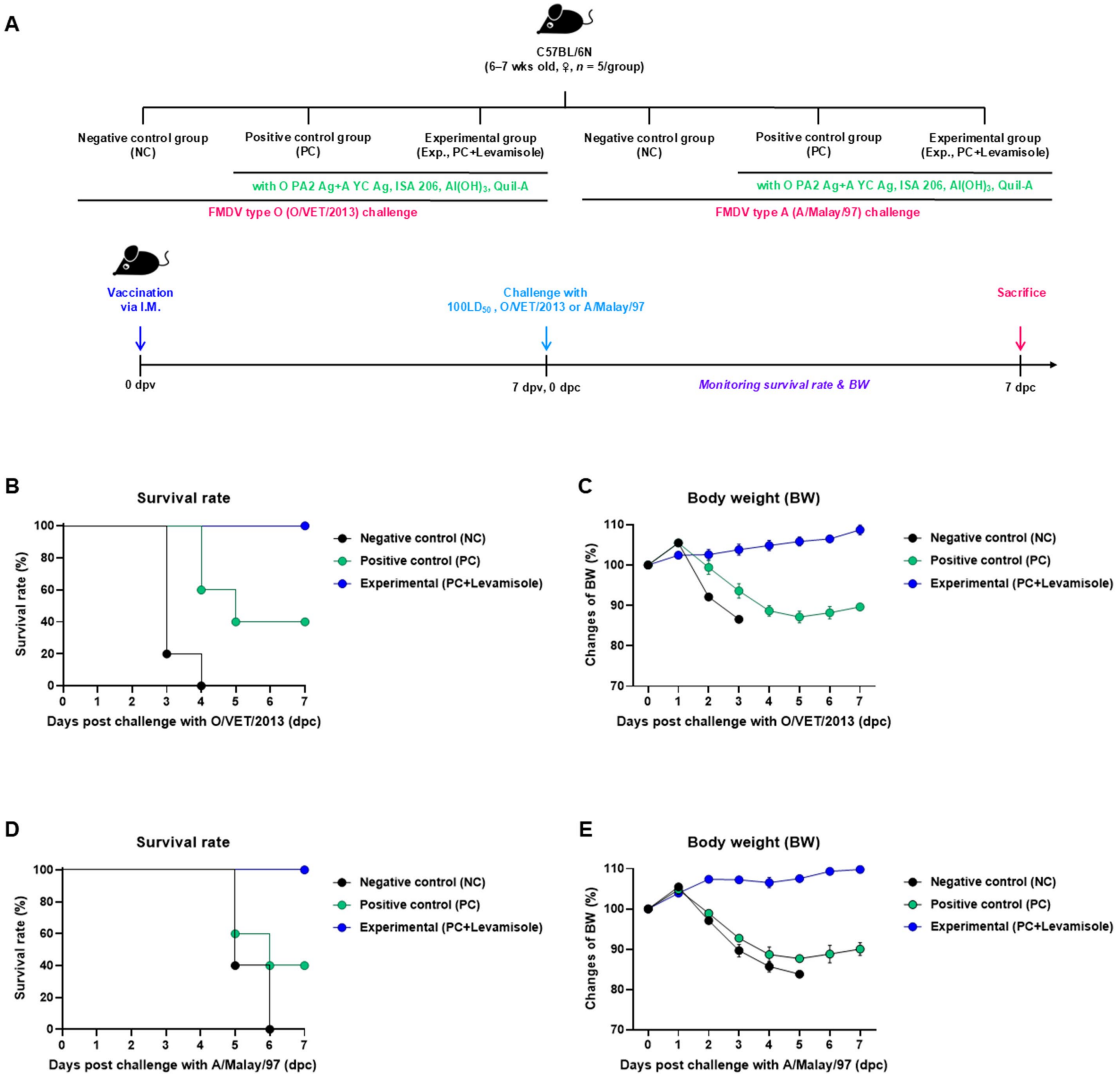
Levamisole enhances the efficacy of FMD vaccine and protects mice against FMDV infection. C57BL/6 mice (*n* = 5/group) were administered foot-and-mouth disease (FMD) vaccine containing inactivated FMD virus (FMDV) type O (O PA2) and A (A YC) antigens (0.375 + 0.375 μg/dose/100 μL; 1/40 of the dose for pigs), 100 μg levamisole/dose/mouse, ISA 206 (50%, w/w), 10% Al(OH)_3_, and 15 μg Quil-A. The positive control (PC) group received the same volume and composition of vaccines as did the experimental (Exp) group but without the addition of levamisole as an adjuvant. The negative control (NC) group was injected with an equal volume of phosphate-buffered saline (PBS). The test vaccines were injected *via* the intramuscular route into mice that were later challenged with FMDV O (100 lethal dose 50%, LD_50_ O/VET/2013) or FMDV A (100 LD_50_ A/Malay/97), at 7 dpv *via* the intraperitoneal route. The survival rates and body weights were monitored for 7 days post-challenge (dpc). **(A–E)** Experimental strategy **(A)**; survival rates post-challenge with O/VET/2013 **(B)** and A/Malay/97 **(C)**; changes in body weight post-challenge with O/VET/2013 **(D)** and A/Malay/97 **(E)**. Data are presented as the mean ± SEM of values from triplicate measurements (*n* = 5/group).

To evaluate the early stages of host defense against FMDV infection rendered by the FMD vaccine containing levamisole as an adjuvant, we performed experiments according to a previously described strategy (Figure 2A). The Exp group administered the FMD vaccine (with the O PA2 + A YC antigen) containing levamisole exhibited a 100% survival rate against FMDV infection (O/VET/2013 or A/Malay/97) (Figures 2B,C). No changes in body weight were observed in the Exp group (Figures 2D,E). The PC group exhibited a 40% survival rate against both FMDV type O and A challenges. The NC group showed a mortality rate of 100% (survival rate of 0%) for both the FMDV type O and A challenges.

### 3.3 FMD vaccine containing levamisole as an adjuvant induces early, mid-term, and long-term immunity in mice

**Figure 3:**
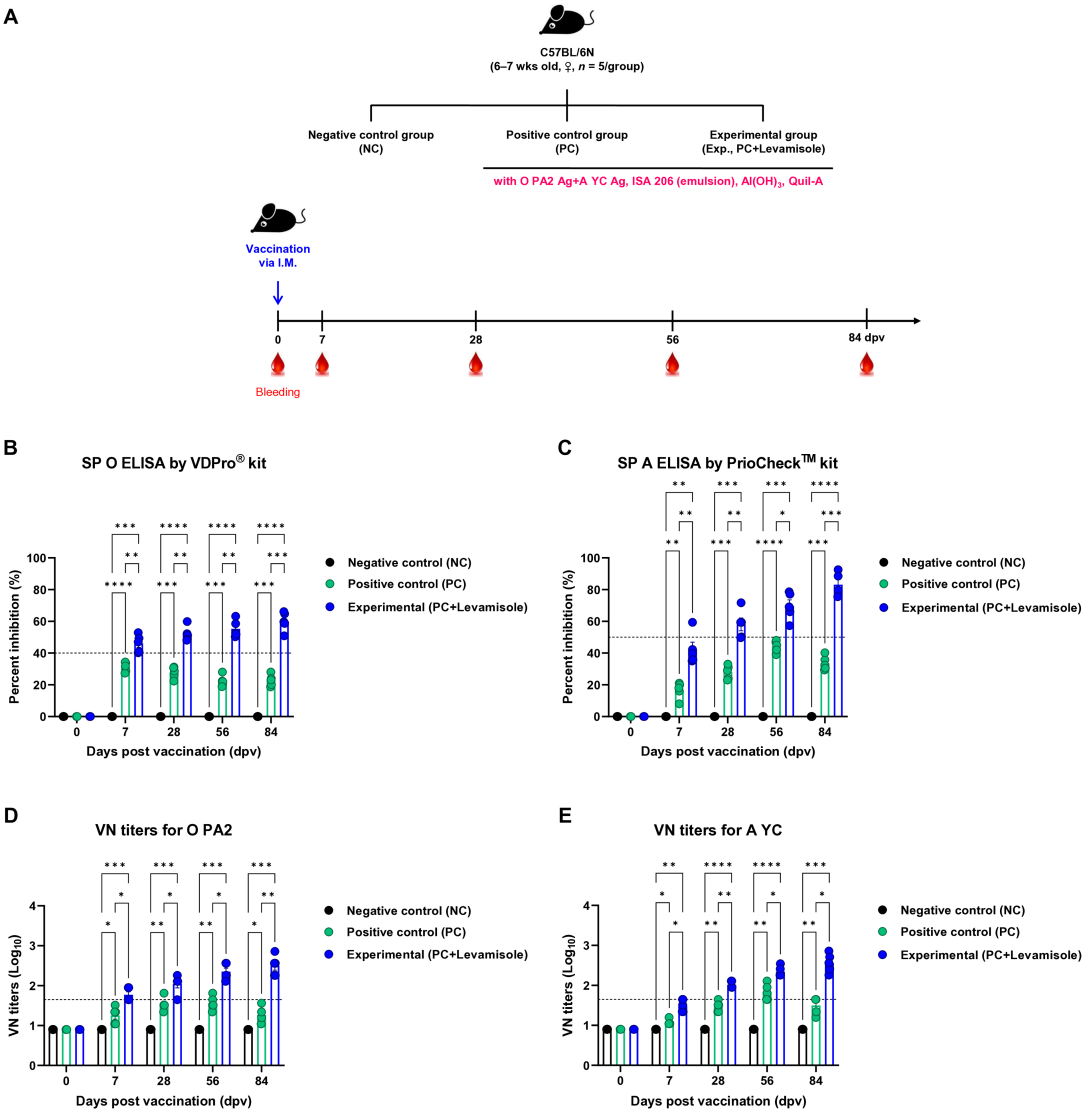
FMD vaccine containing levamisole elicits potent humoral immune response in mice. C57BL/6 mice (*n* = 5/group) were administered foot-and-mouth disease (FMD) vaccine containing inactivated FMD virus (FMDV) type O (O PA2) and A (A YC) antigens (0.375 + 0.375 μg/dose/100 μL; 1/40 of the dose for pigs), 100 μg levamisole/dose/mouse, 50% ISA 206 (w/w), 10% Al(OH)_3_, and 15 μg Quil-A. The positive control (PC) group received the same volume and composition of vaccines as did the experimental (Exp) group but without the addition of levamisole as an adjuvant. The negative control (NC) group was injected with an equal volume of phosphate-buffered saline (PBS). Mice were vaccinated with the test vaccine *via* the intramuscular route, and blood was collected at 0, 7, 28, 56, and 84 days post-vaccination (dpv) for serological analysis using SP O and A ELISA and VN titers for O/PKA/44/2008 (O PA2) and A/SKR/YC/2017 (A YC). **(A–E)** Experimental strategy: **(A)** Ab titers, determined using SP O **(B)** and SP A **(C)** ELISA; and VN titers for O PA2 **(D)** or A YC **(E)** determined using the VN test. Data are presented as the mean ± SEM of values from triplicate measurements (*n* = 5/group). Statistical analyzes were performed using a two-way ANOVA followed by Tukey’s *post-hoc* test. ^*^p < 0.05, ^**^p < 0.01, ^***^p < 0.001, and ^****^p < 0.0001.

To evaluate whether the FMD vaccine with levamisole as an adjuvant induces humoral immunity, we evaluated early, mid-term, and long-term immunity in experimental animals (mice) (Figure 3A). Post-vaccination Ab titers determined using SP O and A ELISA, and VN titers determined using the VN test against O PA 2 and A YC were significantly higher in the Exp group than in the PC group at 7, 28, 56, and 84 dpv. No changes were noted in antibody and VN titers in the NC group (Figures 3B–E).

### 3.4 FMD vaccine containing levamisole as an adjuvant drives early, mid-term, and long-term immunity in pigs

**Figure 4:**
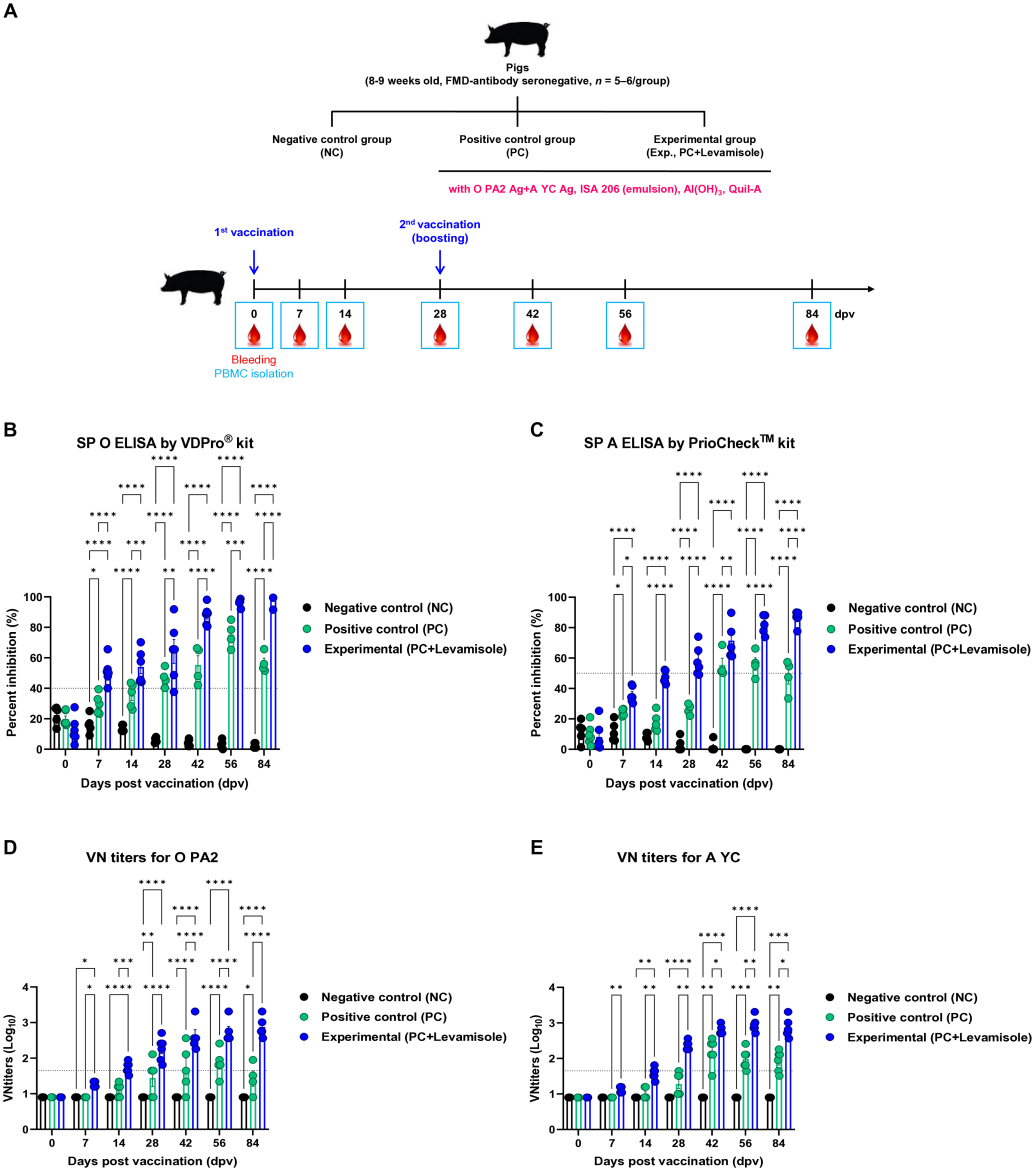
FMD vaccine containing levamisole elicits potent humoral immune response in pigs. Foot-and-mouth disease virus (FMDV) type O and A antibody seronegative pigs (8–9 weeks old) were used. The pigs were divided into three groups (*n* = 5–6/group) and administered inactivated bivalent FMD vaccine without (positive control (PC) group) or with 1 mg/dose/pig levamisole (experimental (Exp) group). The PC group received FMDV type O (O PA2) and type A (A YC) antigens (15 + 15 μg/dose/mL, one dose for cattle and pig use) with ISA 206 (50%, w/w), 10% Al(OH)_3_, and 150 μg Quil-A. Vaccination was performed twice at 28-day intervals, with 1 mL vaccine (one dose) injected *via* the deep intramuscular route into the neck of the animals. The negative control (NC) group was injected with an equal volume of phosphate-buffered saline (PBS). Blood samples were collected from pigs at 0, 7, 14, 28, 42, 56, and 84 days post-vaccination (dpv) for serological assays. **(A–E)** Experimental strategy **(A)**; Abs titers, determined using SP O **(B)** and SP A **(C)** ELISA; and VN titers for O PA2 **(D)** or A YC **(E)**, determined using the VN test. Data are presented as means ± SEM of triplicate measurements (*n* = 5–6/group). Statistical analyzes were performed using two-way ANOVA followed by the Tukey’s *post-hoc* test. ^*^p < 0.05, ^**^p < 0.01, ^***^p < 0.001, and ^****^p < 0.0001.

**Figure 5:**
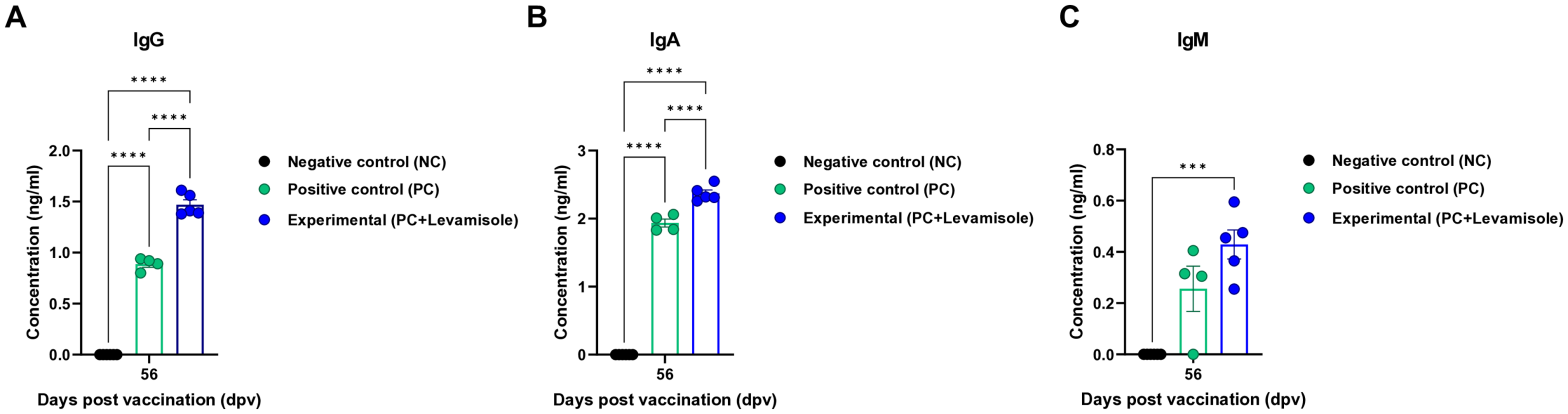
FMD vaccine containing levamisole increases the levels of IgG, IgA, and IgM in pigs. The experimental strategy and method used were the same as those described in the Figure 4 legend. **(A–C)** IgG **(A)**, IgA **(B)**, and IgM **(C)** concentrations. Data are presented as the means ± SEM of triplicate measurements (*n* = 5–6/group). Statistical analyzes were performed using two-way ANOVA followed by the Tukey’s *post-hoc* test. ^***^p < 0.001, and ^****^p < 0.0001.

We evaluated the elicitation of humoral immunity by FMD vaccine containing levamisole as an adjuvant in pigs. Pigs were vaccinated with FMD vaccines containing levamisole, and humoral immunity was evaluated at the early-, mid-term, and long-term stages (Figure 4A). Similar to the results obtained for mice, Ab titers determined using SP O and A ELISA, and VN titers determined using the VN test against O PA 2 and A YC were significantly increased in the Exp group compared with those observed for the PC group at 7, 14, 28, 42, 56, and 84 dpv. No changes were noted in the Ab and VN titers in the NC group (Figures 4B–E). The FMD vaccine containing levamisole also induced a significant increase in immunoglobulin levels. The concentrations of both IgG and IgA were significantly higher in the Exp group than in the PC group at 56 dpv (Figures 5A,B). No significant difference in IgM levels was noted between the Exp and PC groups at 56 dpv (Figure 5C).

### 3.5 FMD vaccine containing levamisole as an adjuvant induces innate and adaptive immunity through the expression of immunoregulatory molecules

**Figure 6:**
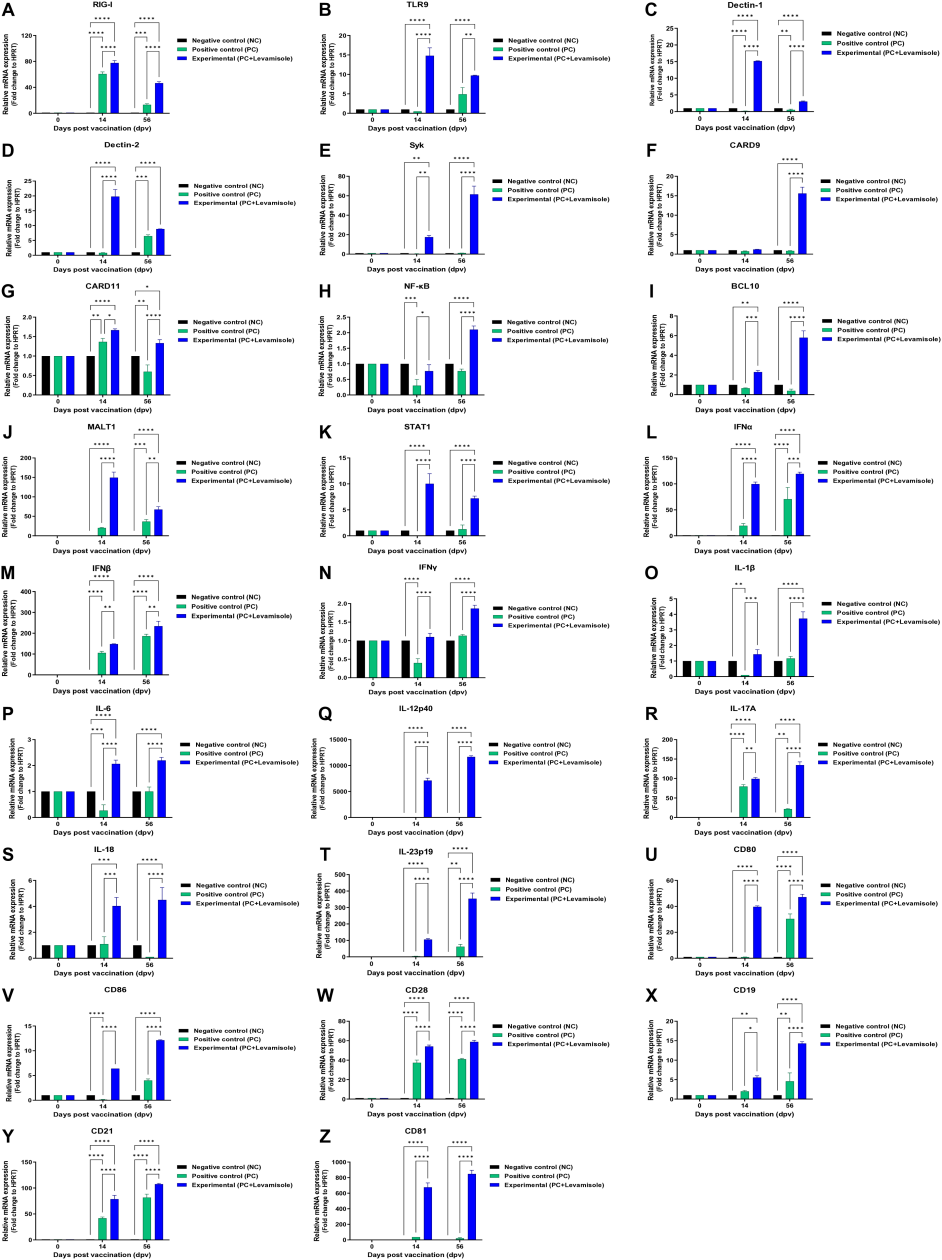
FMD vaccine containing levamisole mediates the expression of immunoregulatory genes in PBMCs from vaccinated pigs. Porcine peripheral blood mononuclear cells (PBMCs) isolated from the whole blood of vaccinated pigs (*n* = 5–6/group), as described in Figure 4A legend, were used for qRT-PCR assays. Gene expression levels were normalized against HPRT levels and are presented as a ratio compared to the control levels. **(A–Z)** Expression levels of RIG-I **(A)**; TLR9 **(B)**; dectin-1 **(C)**; dectin-2 **(D)**; SYK **(E)**; CARD9 **(F)**; CARD11 **(G)**; NF-κB **(H)**; BCL10 **(I)**; MALT1 **(J)**; STAT1 **(K)**; IFNα **(L)**; IFNβ **(M)**; IFNγ **(N)**; IL-1β **(O)**; IL-6 **(P)**; IL-12p40 **(Q)**; IL-17A **(R)**; IL-18 **(S)**, IL-23p19 **(T)**; CD80 **(U)**; CD86 **(V)**; CD28 **(W)**; CD19 **(X)**; CD21 **(Y)**; and CD81 **(Z)**. Data are presented as means ± SEM of triplicate measurements (*n* = 5–6/group). Statistical analyzes were conducted using one-way ANOVA followed by the Tukey’s test. ^*^p < 0.05, ^**^p < 0.01, ^***^p < 0.001, and ^****^p < 0.001.

To understand the mechanism underlying the potent innate and adaptive immune responses induced by the FMD vaccine containing levamisole, we performed qRT-PCR on the selected genes using porcine PBMCs isolated from whole blood samples collected at specific time points (14 and 56 dpv) from pigs vaccinated with the test vaccine (Figures 6A–Z). The expression of retinoic acid-inducible gene (RIG)-I, Toll-like receptor (TLR)9, dendritic cell-associated C-type lectin (dectin)-1, and dectin-2 was significantly increased in the Exp group compared with that in the PC group (Figures 6A–D). The expression of spleen tyrosine kinase (SYK), caspase recruitment domain family member (CARD)9, CARD11, nuclear factor kappa-light-chain-enhancer of activated B cells (NF-κB), B cell lymphoma (BCL)10, mucosa-associated lymphoid tissue (MALT)1, and signal transducer and activator of transcription (STAT) 1 was significantly higher in the Exp group than in the PC group (Figures 6E–K). The expression of INFα, IFNβ, IFNγ, IL-1β, IL-6, IL-12p40, IL-17A, IL-18, and IL-23p19 was significantly increased in the Exp group compared with that in the PC group (Figures 6L–T). The expression of cluster of differentiation (CD)80, CD86, CD21, CD19, CD28, and CD81 was significantly higher in the Exp group than in the PC group (Figures 6U–Z). No significant difference was observed in the gene expression levels in the NC group (Figures 6A–Z).

### 3.6 FMD vaccine containing levamisole induces potent host defenses against FMDV infection in pigs

**Figure 7:**
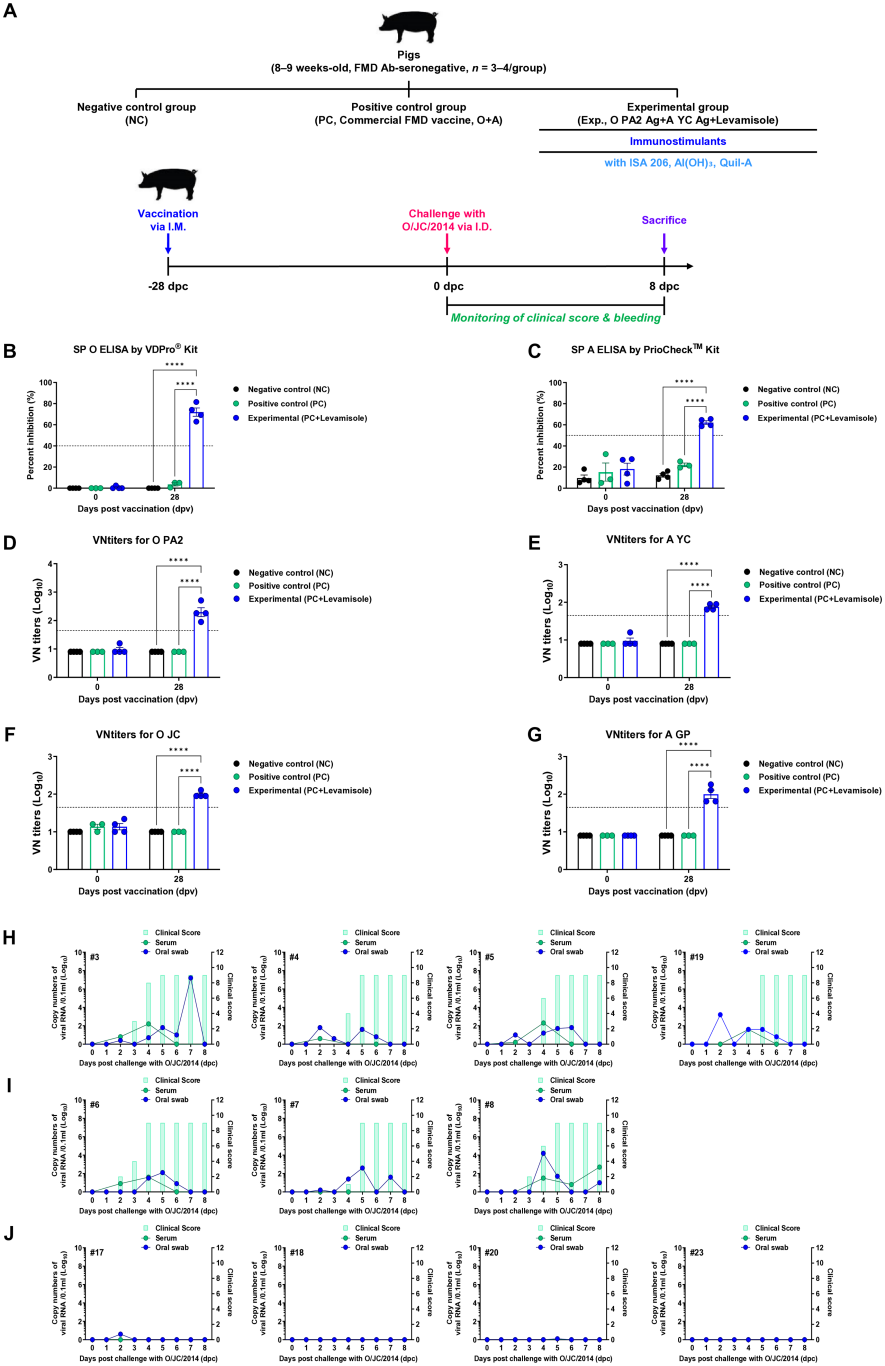
FMD vaccine containing levamisole elicits potent host defense against FMDV infection in pigs. For the challenge experiments, foot-and-mouth disease virus (FMDV) type O and A antibody-seronegative pigs (8–9 weeks old, *n* = 3–4/group) were administered the FMD vaccine containing FMDV type O (O PA2) and type A (A YC) antigens (15 + 15 μg/dose/mL, one dose for cattle and pig use) with levamisole (1 mg/dose/pig), ISA 206 (50%, w/w), 10% Al(OH)3, and 150 μg Quil-A. One-milliliter vaccine was prepared as a single dose and administered to the animals, *via* an intramuscular injection. The positive (PC) and negative (NC) controls were treated with an equal volume of a commercial FMD vaccine (O Primorsky+A Zabaikalski, ARRIAH-VAC^®^; FGBI “ARRIAH”) and phosphate-buffered saline (PBS), respectively, *via* the same route. Blood samples were collected at 0 and 28 days post-vaccination (dpv) for serological assays. Vaccinated pigs were challenged with FMDV type O (O/SKR/JC/2014) on the heel bulb, at a dose of 10^5^ TCID_50_/100 μL, at 28 dpv. **(A–J)** Experimental workflow **(A)**; antibody titers, determined using SP O **(B)** and SP A **(C)** ELISA; VN titers for O PA2 **(D)**, A YC **(E)**, O JC **(F)**, A GP **(G)**, determined using the VN test; and clinical score and viral load in serum samples and oral swabs from the NC (*n* = 4/group) **(H)**, PC (commercial FMD vaccine, *n* = 3/group) **(I)**, and experimental (Exp; O PA2 + A YC + levamisole, *n* = 4/group) groups **(J)** infected with the FMDV type O (O/SKR/JC/2014). The left Y-axis of the graph depicts the viral load in the serum and oral swab samples, represented as log_10_ values, whereas the right Y-axis depicts the clinical index as the maximum value of 10 points. Data are presented as the means ± SEM of triplicate measurements (*n* = 3–4/group). Statistical analyzes were performed using a two-way ANOVA followed by the Tukey’s test. ^****^p < 0.0001.

To assess the efficacy of host defense induced by the FMD vaccine containing levamisole in pigs, a single dose of the vaccine containing levamisole as an adjuvant was administered to the pigs, followed by a challenge with FMDV type O (O JC) at 28 dpv (Figure 7A). The SP O ELISA Ab titers were significantly elevated in the Exp group compared to those in the control groups (PC and NC) (Figure 7B). Similarly, the SP A ELISA Ab titers were higher in the Exp group than in the control groups (PC and NC) (Figure 7C). The time kinetics of the VN titers for O PA2 (Figure 7D) and A YC (Figure 7E) were similar to those of the Ab titers. Notably, the VN titers for O JC (Figure 7F) were higher in the Exp group than in the control groups (PC and NC). Furthermore, the VN titers for A/SKR/GP/2018 (A GP, Figure 7G) were significantly higher in the Exp group than in the control groups. Following the challenge trials, several FMD-related parameters were evaluated, including clinical signs, viremia in serum samples, and viral titers in oral swabs. The NC group (number #3, #4, #5, and #19) infected with FMDV (O JC) exhibited 100% (4/4) typical clinical signs of FMD, with high viral loads detected in both sera and oral swabs (Figure 7H). Similarly, in the PC group (number #6, #7, and #8), all individuals (3/3) displayed clinical signs of FMD, indicating a failure in host protection. Viremia levels in sera and viral shedding in oral swabs for FMDV (O JC) ranged from 1 to 3 log_10_ (Figure 7I). In contrast, the Exp group (number #17, #18, #20, and #23) vaccinated with the FMD vaccine containing levamisole showed no clinical signs of FMD, with no detectable viral titers in the sera or oral swabs (Figure 7J). These results prove that the FMD vaccine with levamisole as an adjuvant elicits potent host defense in pigs.

## 4 Discussion

Extensive research is being conducted to develop vaccines, with a focus on adjuvants (immunostimulants and antigen-delivery systems) and disease-specific antigens, in order to control potential future pandemics of serious infectious diseases (e.g., “infectious disease X”). Immunostimulants include pathogen-associated molecular patterns (PAMPs), damage-associated molecular patterns (DAMPs), and various small-molecules, and antigen-delivery systems include lipid nanoparticles, polymeric nanoparticles, such as PLGA, and caged protein nanoparticles (Zhao et al., 2023). Pattern-recognition receptors (PRRs) are present in the cell membrane or cytoplasm and mediate innate immunity by recognizing PAMPs and DAMPs. The PRR signaling pathway is well-characterized as an initiator of this cascade (Tang et al., 2012; Zindel and Kubes, 2020; Ma et al., 2024). Among the numerous small-molecules, levamisole had previously been identified as a vaccine adjuvant based on its ability to modulate the host immune system (Brunner and Muscoplat, 1980; Gholami et al., 2023). It is used in several countries for various veterinary diseases, and with the emergence of the COVID-19 pandemic, it has been proposed as a treatment strategy for improving the clinical condition of patients with COVID-19 (Shomali et al., 2012; Sultana et al., 2020; El Khatabi et al., 2022). The World Health Organization has set the acceptable daily intake of levamisole to 0.006 mg/kg body weight. The US approved the use of levamisole in cattle in 1978 (NADA 102–437) (Krautmann et al., 2023). Levamisole has a safety margin of 3X in relation to its safe use in ruminants (Bogan et al., 1982; Fernández et al., 1997).

In this study, no cytotoxic effects of levamisole on murine PECs and porcine PBMCs were noted at 0–5 μg/mL concentrations. Levamisole effectively induced the expression of IFNγ in murine PECs and porcine PBMCs, and when administered with antigens, it further increased the expression of IFNγ (Figure 1). Based on these results, levamisole can potentially be used as an adjuvant. Levamisole promotes the activation of murine bone marrow-derived DCs and induces Th1 immune responses *in vitro* and *in vivo* (Fu et al., 2016). Given that cytokine secretion was confirmed 24 h after treating cells with levamisole with or without antigens, IFNγ can be considered to have been secreted by innate immune cells, such as natural killer (NK) cells, in PECs and PBMCs, rather than through the stimulation of cellular immunity, such as the Th1 response. Considering the fact that levamisole exhibits antiviral action by inducing the secretion of type I IFN (Muñoz et al., 1986; Ruiz-Moreno et al., 1993), we evaluated whether levamisole itself elicits a host defense response against FMDV infection. However, levamisole alone, without an antigen, did not show a host-protective effect at 3 or 5 dpi. As previously mentioned, owing to the short half-life of levamisole, partial host defense might have been observed under FMDV challenge before 3 dpi. However, the main objective of this study was to evaluate the potential of levamisole as an immunostimulant and not to assess its effect as an antiviral agent. We did not aim for an initial protection within 3 days, regardless of whether antibodies or neutralizing antibodies were produced after receiving the commercial vaccine. Because the sustainability of vaccine-mediated immunity is important, we presumed that the clinical application of levamisole would be difficult if the protection was not sustained post the initial 3 days. Moreover, we considered the use of levamisole together with vaccines in view of its antiviral effect. The test vaccine, containing levamisole as an adjuvant, showed early host defense against FMDV infection in experimental animals (mice) (Figure 2) and elicited early, mid-term, and long-term immunity (Figure 3). It also induced a potent adaptive immune response (Figure 4) and significantly increased the immunoglobulin levels (Figure 5) in the target animals (pigs). These results indicated that levamisole effectively induced not only initial immunity, but also conferred long-lasting immunity, proving its potential as a novel adjuvant.

To elucidate how levamisole effectively induces innate and adaptive immunity, the expression of immune-related genes was confirmed using whole blood-derived PBMCs from pigs immunized with the levamisole-containing FMD vaccine (Figure 6). In this study, the vaccine containing levamisole effectively induced STAT1 and NF-κB expression by upregulating the CARD9 or 11–BCL10–MALT1 (CBM) complex. The expression of cytokines, such as IFNα, IFNβ, IFNγ, IL-1β, IL-6, IL-12p40, IL-17A, IL-18, and IL-23p19, costimulatory molecules (CD80 and CD86), T-cell receptors (CD28), and B-cell core receptors (CD19, CD21, and CD81) was significantly increased. The FMD vaccine containing levamisole stimulated RIG-I, TLR9, dectin-1, and dectin-2 in porcine PBMCs. The stimulation of C-type lectin receptors (CLR), such as dectin-1, dectin-2, mincle, and DC-SIGN, plays a pivotal role in the early stages of host defense during pathogen infection by activating the host innate immune system (Zhao et al., 2023). Stimulation of RIG-I induces the expression of type I IFN (IFNα and IFNβ), through the expression of interferon regulatory factor (IRF)3 and elicits Th1 responses, cross presentation, and CTL responses (Cao et al., 2024). The activation of Dectin-1 and Dectin-2 promotes NF-κB expression through their downstream signaling pathways (Syk and CBM complex), leading to the secretion of proinflammatory cytokines such as IL-1β, IL-6, IL-12p40, IL-23p19, and IL-17A (LeibundGut-Landmann et al., 2007; Gringhuis et al., 2009). These cytokines play a crucial role in both innate and adaptive immune responses by inducing the activation of Th1, Th17, and cytotoxic T cells, as well as antibody-mediated immunity (Malamud and Brown, 2024). IFNγ, a type II interferon, is primarily secreted by Th1, cytotoxic T, and NK cells. It serves as a key proinflammatory cytokine that regulates the functions of various immune cells, including T and B cells (Billiau and Matthys, 2009). IFNγ, along with STAT1, upregulates the expression of T-bet, a critical regulator that stabilizes the Th1 phenotype, thereby contributing to T cell-mediated immunity (Afkarian et al., 2002; Cope et al., 2011). IFNγ also promotes the expression of CD40, CD80, and CD86 molecules on DCs, facilitating their maturation (Xue et al., 2013; Liu et al., 2022). The T cell receptor CD28 interacts with various ligands (CD80 and CD86) to support the sustained differentiation and proliferation of T cells (Bour-Jordan et al., 2011; Esensten et al., 2016). CD21, expressed on the surface of B cells, forms a complex with CD19 and CD81, regulating B-cell-mediated humoral immune responses (Tedder et al., 1994; Tedder et al., 1997). In conclusion, the FMD vaccine containing levamisole stimulates various PRRs and activates their downstream signaling pathways, enhancing the production of pro-inflammatory cytokines. These cytokines promote the maturation and differentiation of diverse immune cells (DCs, macrophages, and T and B cells), thereby driving robust innate and adaptive immune responses.

The test vaccine containing levamisole elicited 100% host protection against FMDV type O infection in pigs (Figure 7). When vaccinated with levamisole, innate-like cells (γδ T, invariant NK T (iNKT), and mucosal-associated invariant T cells) are activated after the secretion of IL-23 from APCs, and IL-17A is secreted from these cells. Neutrophil extracellular traps (NETs) and macrophage extracellular traps (METs) are formed during viral infection through the recruitment and training of neutrophils and macrophages *via* the IL-23/IL-17A axis, which is believed to critically contribute to host defense by inducing NETosis and METosis in pathogens. Unlike adaptive immunity, innate immunity has no memory response. However, recent studies (Netea et al., 2016; Netea et al., 2019; Stothers et al., 2021; Thiem et al., 2021; Mu et al., 2022; Cheong et al., 2023) have reported that innate immune cells form an immune memory to a primary infection and then induce a potent immune response to a secondary infection. This immune response is called the innate immune memory. Sherwood et al. (2022) reported a response to host innate immune memory. Innate immune memory is elicited through activation of PRRs and cytokine action on immune cells. Additionally, immune cells trained through primary infection are induced and maintained by reprogramming their metabolism and transcriptional patterns. These functions promote the host response to infection by enhancing leukocyte expansion, phagocytosis, and pathogen killing. However, in this study, only gene expression levels were measured due to the lack of commercial cytokine detection kits for pigs and antibodies for western blot. In future studies, we plan to overcome these shortcomings by elucidating the background of the robust immune response induced by levamisole at the protein level during the extensive vaccination period. Based on these results, it can be inferred that the FMD vaccine with levamisole sequentially activates immune-related signaling pathways through the activation of PRRs, elicits potent innate and diverse adaptive immunity in experimental and target animals, and ultimately protects the host. Collectively, our findings should contribute to the development of a future FMD vaccine that overcomes several limitations of commercial FMD vaccines and provides a novel strategic vaccine platform for controlling livestock diseases that pose serious problems.

## Data Availability

The original contributions presented in the study are included in the article/Supplementary material, further inquiries can be directed to the corresponding author.
