# Supplementary material for: Levamisole, as a viral vaccine adjuvant, induces robust host defense through the modulation of innate and adaptive immune responses
Source: Front Microbiol. 2025 Jan 8;15:1493561. doi: 10.3389/fmicb.2024.1493561 (PMC11751227; doi:10.3389/fmicb.2024.1493561)
Supplement: Supplementary file 1 [file Data_Sheet_1.pdf]

Supplementary Table legends

Supplementary Table 1. List of primer sequences for qRT-PCR.

Supplementary Figure legends

Supplementary Figure 1. Cytotoxicity of Levamisole measured in BHK-21, LF-BK, ZZ-R, Murine PECs and Porcine PBMCs *via* cell viability assay.

Cell viability of BHK-21 cells (**A**), LF-BK cells (**B**), ZZ-R cells (**C**), Murine PECs (**D**), and Porcine PBMCs (**E**)

The data represent the mean ± SEM of triplicate measurements. Statistical analyses were performed using two-way ANOVA with Tukey test.

Supplementary Figure 2. Assessment of induction of host defense against FMDV infection 3 days and 7 days after inoculation with levamisole alone in mice

Levamisole alone was injected intramuscularly into C57BL/6 mice that were challenged with FMDV O (100 LD_{50} O/VET/2013) or FMDV A (100 LD_{50} A/Malay/97) 3 days post-inoculation (dpi) and 7 dpi by intraperitoneal injection. Survival rates and body weights were monitored for 7 days post-challenge (dpc) with the respective viruses. (**A**) Experimental workflow, survival rates post-challenge with (**B**) O/VET/2013 or (**C**) A/Malay/97, and changes in body weight post-challenge with (**D**) O/VET/2013 or (**E**) A/Malay/97 at 3 dpv; survival rates post-challenge with (**F**) O/VET/2013 or (**G**) A/Malay/97, and changes in body weight post-challenge with (**H**) O/VET/2013 or (**I**) A/Malay/97 at 7 dpi. Data are presented as mean ± SEM of triplicate measurements (n = 5/group).

Supplementary Table 1

Target	Primer name	Sequence (5'- 3')	Length (mer)
RIG-I	RIG-I F	GCACCTCATACTTACAGCCCA	21
	RIG-I R	CCACAACCAGTAGGAGCACAT	21
TLR9	TLR9 F	TCCTCTACGACTGCATCACCA	21
	TLR9 R	GTAATTGAAGGACAGGTTGAGCTT	24
Dectin-1	Dectin-1 F	ACAGCTCCAAAGAGCTGGAA	20
	Dectin-1 R	CCAGCTCTTTGGAGCTGTCTA	20
hDectin-2	hDectin-2 F	GCTGAGTCTCTGGGCAACAT	20
	hDectin-2 R	TGAGGTTGCTGCTCTTGCAT	20
SYK	SYK F	CCAACCACTTGCCCTTCTTC	20
	SYK R	ATGGTGTAGTGATGCGCCTT	20
CARD9	CARD9 F	CCGCAGCTCTACAAGAAGGT	20
	CARD9 R	TCTGCAGCTTCATCACCTCG	20
hCARD11	CARD11 F	TGAACGAGGTCATCAAGCTG	20
	CARD11 R	AGCGTCAGCTGCTTCTTCTC	20
NF-κB	NF-κB F	TCGCTGCCAAAGAAGGACAT	20
	NF-κB R	AGCGTTCAGACCTTCACCGT	20
BCL10	BCL10 F	ATGGAGCCCGCCGCGCCGTC	20
	BCL10 R	GCTATGATTTTTTCACACAG	20
MALT1	MALT1 F	GTTGGAAGCCCCATTCCACA	20
	MALT1 R	ACTCCACTGCCTCATCTGTTC	21
STAT1	STAT1 F	TGCACGATGGTCTCAGCTTT	20
	STAT1 R	CAGCAGTGGGACCAAGAAGT	20
IFNα	IFNα F	CATCTGCTCTCTGGGCTGTG	20
	IFNα R	TGAGGGGATCCAAAGTCCCT	20
IFNβ	IFNβ F	TGCAACCACCACAATTCCAGA	21
	IFNβ R	GGTTTCATTCCAGCCAGTGC	20
IFNγ	IFNγ F	GCCATTCAAAGGAGCATGGAT	21
	IFNγ R	CTGATGGCTTTGCGCTGGAT	20
IL-1β	IL-1β F	AGCCAGTCTTCATTGTTCAGGT	22

	IL-1β R	TCATCTCTTTGGGGCCATCAG	21
IL-6	IL-6 F	CTGCAGTCACAGAACGAGTG	20
	IL-6 R	CGGCATCAATCTCAGGTGCC	20
IL-12p40	IL-12p40 F	GGAGTATAAGAAGTACAGAGTGG	23
	IL-12p40 R	GATGTCCCTGATGAAGAAGC	20
IL-17A	IL-17A F	CTCGTGAAGGCGGGAATCAT	20
	IL-17A R	GGTGTGCTCCGGTTCAAGAT	20
IL-18	IL-18 F	AGCTGAAAACGATGAAGACCTG	22
	IL-18 R	AAACACGGCTTGATGTCCCT	20
IL-23p19	IL-23p19 F	CCATATCCAGTGCGGGGATG	20
	IL-23p19 R	AGGCCTTGGTGGATCCTTTG	20
CD80	CD80 F	TCAGACACCCAGGTACACCA	20
	CD80 R	GACACATGGCTTCTGCTTGA	20
CD86	CD86 F	TTTGGCAGGACCAGGATAAC	20
	CD86 R	GCCCTTGTCCTTGATTTGAA	20
CD28	CD28 F	TCAAAGGAGTTCCGGGCATC	20
	CD28 R	CTGAAGCAGGCGGGAGTAAT	20
CD19	CD19 F	GGACGACAGACTTCCTGAGC	20
	CD19 R	GTTCTGGCCCATCAGGATTA	20
CD21	CD21 F	TGCCATGCCTACAAAGCTGA	20
	CD21 R	GTAGTAACCAGGGCGGCATT	20
CD81	CD81 F	TCAACAAGGACCAGATCGCC	20
	CD81 R	GAGCGTCTCGTGGAAAGTCT	20
HPRT	HPRT F	CCCAGCGTCGTGATTAGTGA	20
	HPRT R	GCCGTTCAGTCCTGTCCATA	20

Supplementary Figure 1

(A)

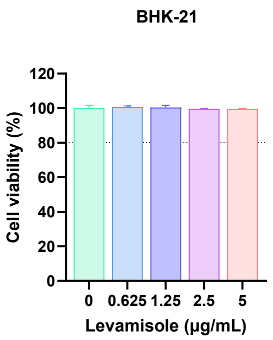

(B)

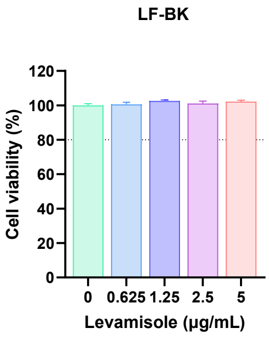

(C)

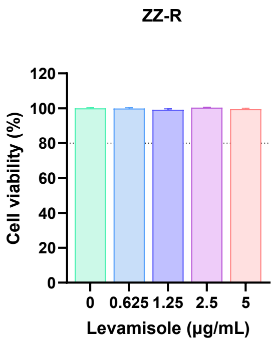

(D)

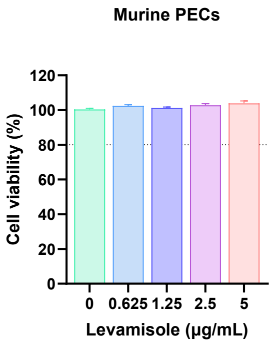

(E)

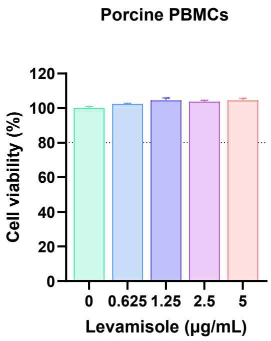

Supplementary Figure 2

(A)

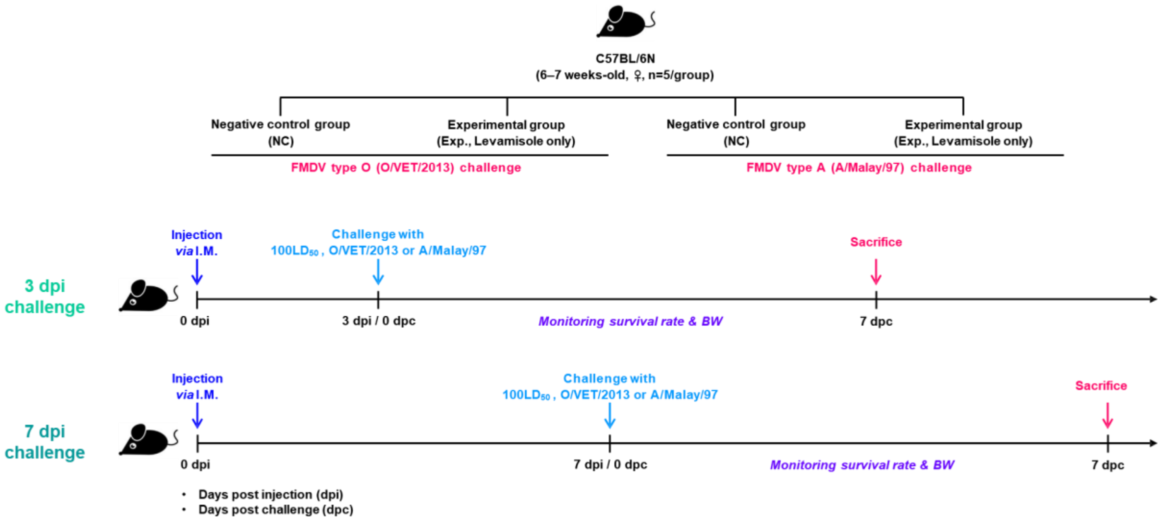

(B)

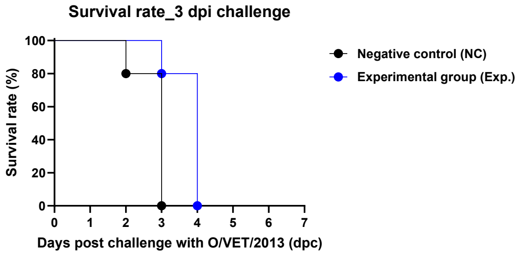

(C)

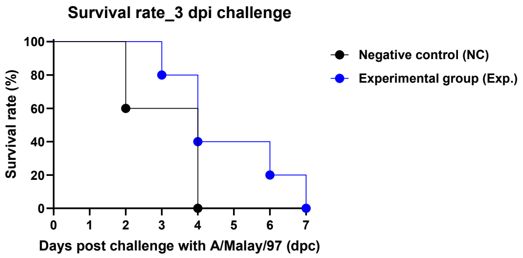

(D)

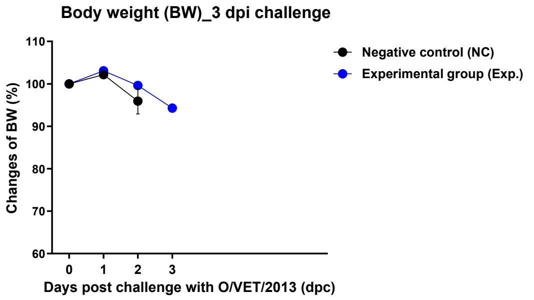

(E)

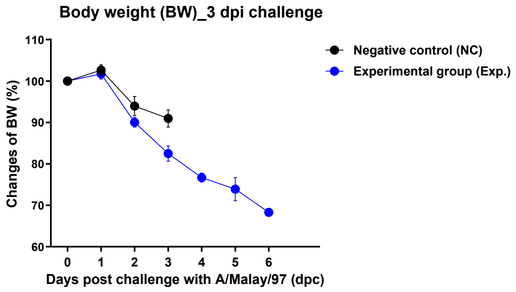

(F)

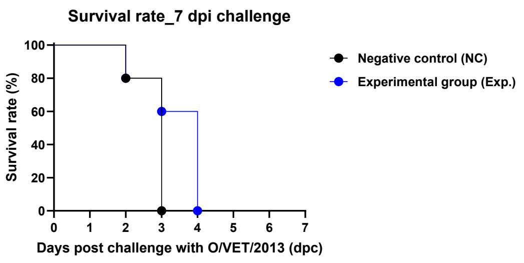
Survival rate_7 dpi challenge
Survival rate (%)
100
80
60
40
20
0
0 1 2 3 4 5 6 7
Days post challenge with O/VET/2013 (dpc)
Negative control (NC)
Experimental group (Exp.)

(G)

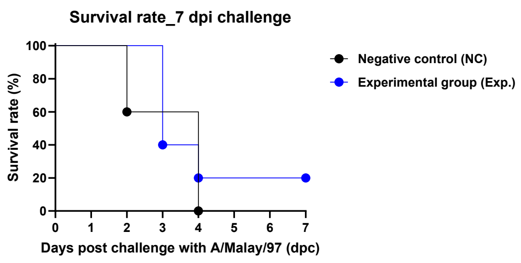
Survival rate_7 dpi challenge
Survival rate (%)
100
80
60
40
20
0
0 1 2 3 4 5 6 7
Days post challenge with A/Malay/97 (dpc)
Negative control (NC)
Experimental group (Exp.)

(H)

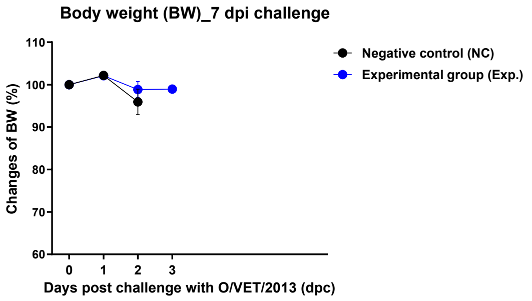
Body weight (BW)_7 dpi challenge
Changes of BW (%)
110
100
90
80
70
60
0 1 2 3
Days post challenge with O/VET/2013 (dpc)
Negative control (NC)
Experimental group (Exp.)

(I)

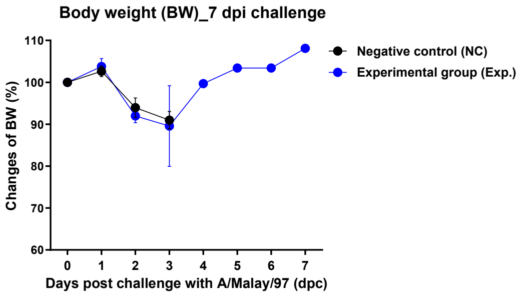
Body weight (BW)_7 dpi challenge
Changes of BW (%)
110
100
90
80
70
60
0 1 2 3 4 5 6 7
Days post challenge with A/Malay/97 (dpc)
Negative control (NC)
Experimental group (Exp.)
